# Photo-Reduction of CO_2_ by VIS Light on Polythiophene-ZSM-5 Zeolite Hybrid Photo-Catalyst

**DOI:** 10.3390/molecules24050992

**Published:** 2019-03-12

**Authors:** Jana Kianička, Gabriel Čík, František Šeršeň, Ivan Špánik, Robert Sokolík, Juraj Filo

**Affiliations:** 1 Department of Environmental Engineering, Faculty of Chemical and Food Technology, Slovak University of Technology in Bratislava, Radlinského 9, SK-812 37 Bratislava, Slovakia; jana@kianicka.name; 2 Institute of Chemistry, Faculty of Natural Science, Comenius University in Bratislava, Ilkovičova 6, SK-842 15 Bratislava, Slovakia; robert.sokolik@uniba.sk; 3 Department of Analytical Chemistry, Faculty of Chemical and Food Technology, Slovak University of Technology in Bratislava, Radlinského 9, SK-812 37 Bratislava, Slovakia; ivan.spanik@stuba.sk

**Keywords:** carbon dioxide, VIS light photo-catalysis, polythiophene, ZSM-5, spin-trapping

## Abstract

A new hybrid photo-catalyst based on ZSM-5 zeolite suitable for reduction of carbon dioxide was synthesized. The photo-catalyst was prepared by oxidative polymerization of thiophene with FeCl_3_ in the presence of ZSM-5 with participation of ultrasound. The synthesized photo-catalyst strongly absorbs light radiation up to approx. 650 nm, with the absorption edge in the NIR region. Reactive radicals were generated by VIS light irradiation in an aqueous suspension consisting of the photo-catalyst with CO_2_. Formic acid and acetic acid were generated as the main products of the CO_2_ reduction. EPR spin trapping technique was applied to identify the reactive radical intermediates. In this work, the mechanism of product formation is also discussed.

## 1. Introduction

The concentration of one of the most important greenhouse gases, carbon dioxide, is steadily increasing. According to the NOAA (National Centers for Environmental Information) [1] the CO_2_ concentration rises approximately 2 ppm every year. In March 2014, the concentration of carbon dioxide broke the 400 ppm milestone [2]. This negative situation results in climate changes and global warming. The average temperature in August 2015 was 0.88 °C above the 20th century average of 15.6 °C and overcame the 136-year record [1]. 

Decreasing CO_2_ emission from the atmosphere through pre- and/or post-combustion and through capturing and sequestration is energy demanding, thus uneconomical [3]. On the other hand, the light-driven reduction of carbon dioxide is one of the most promising solutions for the possibility of storing solar energy, since CO_2_ is reduced at relatively low temperature and atmospheric pressure to give added value compounds [3,4]. Looking for an environmental-friendly photo-catalyst for effective reduction of CO_2_ with H_2_O is the most challenging goal of many studies today. There are many thoroughly researched inorganic semiconducting materials such as TiO_2_, CdS, ZnO, WO_3_, GaP and others [5]. Inoue et al. [6] in 1979 demonstrated for the first time the photo-catalytic reduction of CO_2_ with water on TiO_2_ to give formic acid, formaldehyde, methanol, and methane as the main products. Improvements to these systems were made because the recombination of the photo-induced pair electron-hole was very fast and the catalyst absorbed only a small fraction of light in the UV region of the solar spectrum (no absorption was observed in the visible region) [7]. These disadvantages can be overcome by doping of semiconductors with different metal, cations and ions (Fe^3+^, Mo^5+^, Ru^3+^, Os^3+^, Re^5+^, V^4+^, Rh^3+^, anions from C, N, F, P, S); coupling with other semiconducting oxides (Cu_2_O, PbS, CdS, CdSe) and sensitization by dye molecules (erythrosin B, eosin). The band gap can be reduced by suitable modification and the photo-reactivity for both oxidation and reduction can be increased [7]. 

The photo-catalytic activity of the TiO_2_ semiconductor can drastically increase as the particle sizes became smaller than 10 nm due to charge carrier mobility [8]. Crystalline microporous aluminosilicates—zeolites—with cages and channels of molecular dimensions are very suitable for this purpose. Cavities create conditions for stabilization of metastable species, providing photochemical routes that are typically unfavorable in other media. In the building units of TO_4_ (T = Al, Si) aluminum causes a charge imbalance which is balanced by the extra framework of the ion-exchangeable cations. Due to their properties, zeolites are widely used in environmental, consumer, chemical, and petrochemical industries [9]. In order to further reduce the particle sizes of TiO_2_ photo-catalysts, highly dispersed titanium oxide clusters were prepared in microporous zeolite Y and molecular sieves. UV activation of the TiO_2_-mesoporous zeolites, Ti-MCM-48 and Ti-MCM-41 leads to the photo-catalytic reduction of CO_2_ and H_2_O and formation of CH_3_OH and CH_4_ as main products. Dispersed titanium oxide clusters confined in zeolite micropores showed significantly higher activity in CO_2_ reduction than the powdered TiO_2_ catalyst [10].

Another possibility for charge transport is use of organic dyes or organic semiconductors, which often possess better optical, electronic, chemical, and structural properties compared to inorganic semiconductors. Polythiophene (PT) is a very important part of conjugated polymers, where sp^2^pz hybridization causes there to be one unpaired electron in the carbon atom and thereby possesses properties of semiconductors or metals [11]. PT without side chains is neither soluble nor fusible and thus PT has been widely used in chemical and optical sensors, light-emitting diodes and displays, photovoltaic devices, and solar cells since 1980 [12]. In our earlier work, we prepared a heterogeneous photo-catalytic system (thiophene oligomers—ZSM-5 zeolite) suitable for photolytic degradation of organic pollutants such as 4-chlorophenol by visible light [13]. Inspired by the polythiophene properties, here we are interested in the preparation of environmental-friendly photo-catalysts suitable for photo-reduction of CO_2_ into value added compounds. The goal of this study was the preparation of the photo-catalyst Na-ZSM-5-PT by chemical oxidative polymerization of thiophene for CO_2_ photo-reduction. The synthesized photo-catalyst was characterized by UV-Vis spectroscopy, FT-IR spectroscopy, SEM (scanning electron microscopy), Mössbauer spectroscopy, and thermal gravimetric analysis. The photo-catalytic activity of the material was evaluated. Finally, a mechanism for the photo-catalytical reduction of CO_2_ was suggested. 

## 2. Results and Discussion

### 2.1. Characterization of Hybrid Photo-Catalyst Na-ZSM-5-PT

An accompanying feature in the preparation of photo-catalysts, after the addition of thiophene, was a color change from the light brown (from FeCl_3_) aqueous suspension and Na-ZSM-5 to brown until black. Color changes also occurred in the prepared photo-catalysts, from the original white to brown to black shades in hybrid systems based on the Na-ZSM-5 zeolite (See Figures S1–S3 in supporting information). In our previous work [14], we found that by irradiation of zeolite ZSM-5, whose crystals were on the surface covered by a layer of organic semiconductor (polythiophene), with visible light resulted in the production of reactive forms of oxygen (ROS) which effectively kills pathogenic microorganisms in an aquatic environment. By using the EPR (electron paramagnetic resonance) spin trapping technique and applying 5,5-dimethyl-1-pyrroline-*N*-oxide (DMPO) as a spin trapping agent we demonstrated that the effective “killing” particle in the microorganisms was a hydroxyl radical, detected as ^•^DMPO–OH spin-adduct under the given experimental conditions. 

**Figure 1:**
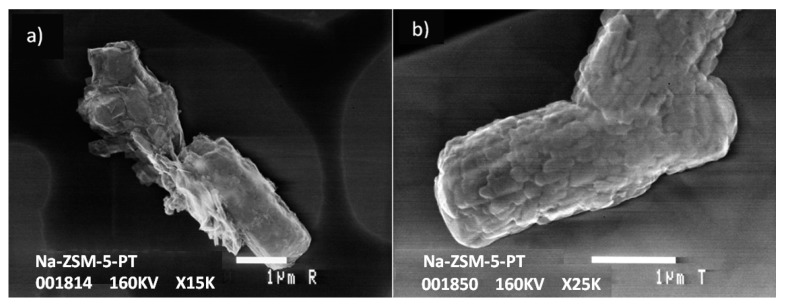
SEM images of the synthesized photo-catalysts Na-ZSM5-PT: (**a**) without the use of ultrasound (**b**) with ultrasound participation.

Furthermore, polythiophene, generated by the oxidative polymerization of thiophene with FeCl_3_, was not imposed evenly on the crystal surface, but produced non-uniform polymer agglomerates, which greatly distorted the “uniform surface” of the zeolite crystals. To prevent this effect to the greatest extent possible, we performed the polymerization of thiophene with participation of ultrasonic stirring. It has been shown that this technique also leads to effective polymerization, in which case the crystals of ZSM-5 were uniformly coated by the formed polythiophene across the whole surface. Figure 1a shows the surface of the hybrid catalyst, prepared by oxidative polymerization, but the reaction mixture was effective stirred only with a magnetic stirrer. Figure 1b shows a SEM image of the synthesized hybrid photo-catalyst, obtained by the ultrasound participation (the reaction flask was immersed in a water bath with the ultrasound; bath temperature was stabilized at 50 ± 2 °C). The comparison of the two images clearly shows that the effect of the ultrasonic stirring is reflected in a uniformly cascade-deposition of thin polymer layers across the whole surface of the zeolite crystal. It can be assumed that the photo-catalyst prepared by the ultrasound participation ensures a higher specific surface area and thus a more efficient supply of oxygen and reactive substrate molecules (CO_2_) to the crystal surface of the photo-catalyst.

**Figure 2:**
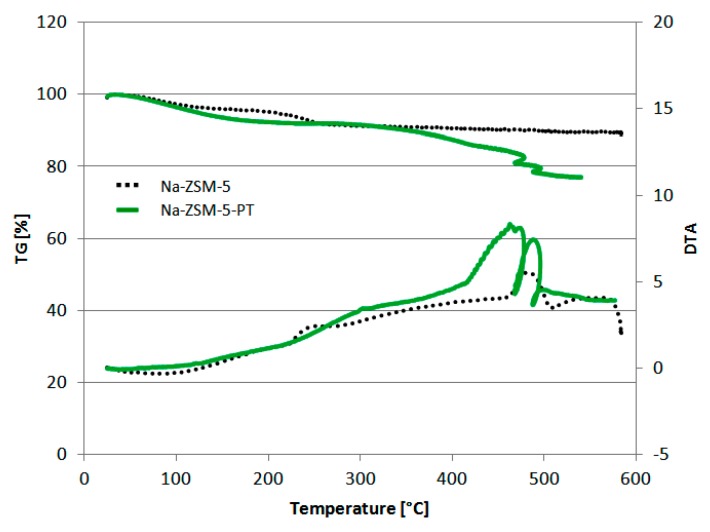
TG/DTA analysis of pure zeolite (Na-ZSM-5) and the hybrid system Na-ZSM-5-PT.

Figure 2 shows the TG/DTA curves of pure Na-ZSM-5 and the synthesized hybrid photo-catalyst Na-ZSM-5-PT. From the TGA curves it can be seen that in both systems, there was only a slight weight loss up to 100 °C, which may be attributed to the evaporation of the bound water or evaporation of the residual thiophene monomer and its lower oligomers in the hybrid photo-catalyst, respectively. The weight loss in Na-ZSM-5-PT is at 100 °C ca. 3 wt%. According to the DTA curves, oxidative degradation of the polymer in the oxygen atmosphere is observable at 350 °C. Two exothermic maximums at about 475 °C and 490 °C can be registered on the DTA curve of hybrid Na-ZSM-5-PT. Both of these peaks are of course subject to the thermal decomposition process of the present organic materials in the zeolite structure. While the first peak (475 °C) is clearly related to the oxidation of polythiophene, the second (490 °C) is more likely associated with the thermal oxidation reactions of the remaining organic materials (low concentrations) which are always added to the reaction mixture for the synthesis of ZSM-5. These materials fulfill the function of structure directing agents—SDAs (“templates”)—in the formation of a concrete zeolite structure [15]. This idea is also supported by the fact that at an approximately equal temperature (490 °C) the maximum on the DTA curve of non-modified zeolite also appears.

Based on these results, we can conclude that “regularly deposited” layer of organic semiconductor (PT) on the surface of the crystal Na-ZSM-5 provides sufficient thermal stability during photocatalyzed reactions with the catalyst Na-ZSM-5-PT.

**Figure 3:**
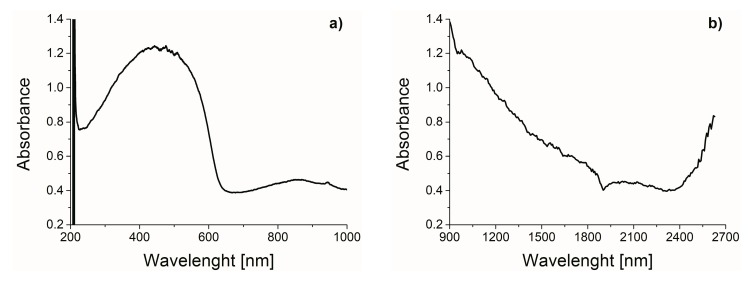
UV–VIS reflectance spectrum (displayed in absorbance) of synthesized photo-catalyst in the wavelength range 200–1100 nm (**a**) and a NIR spectrum in the range 900–2000 nm (**b**).

UV–VIS spectrum of the photo-catalyst is shown in Figure 3a. The curve illustrates that the maximum of the absorption band is at wavelength ca. 480 nm. Pure PT has a maximum at 476 nm [16]. The record also shows that on the base polymer backbone there are present cation radicals (PT^•+^) and anion radicals (PT^•−^) (polarons) [17] shifting the absorption into the infrared region. Figure 3b shows the NIR spectrum of heterogeneous system. The absorption in the red and near-infrared region is proof of the formation of dications (bipolarons) during the polymerization [18]. 

The photo-catalyst was characterized also by FT-IR spectroscopy. Spectra (See Figures S4 and S5 in supporting information) were recorded before and after photo-reaction to monitor the photo-catalyst stability. No significant changes were observed.

**Figure 4:**
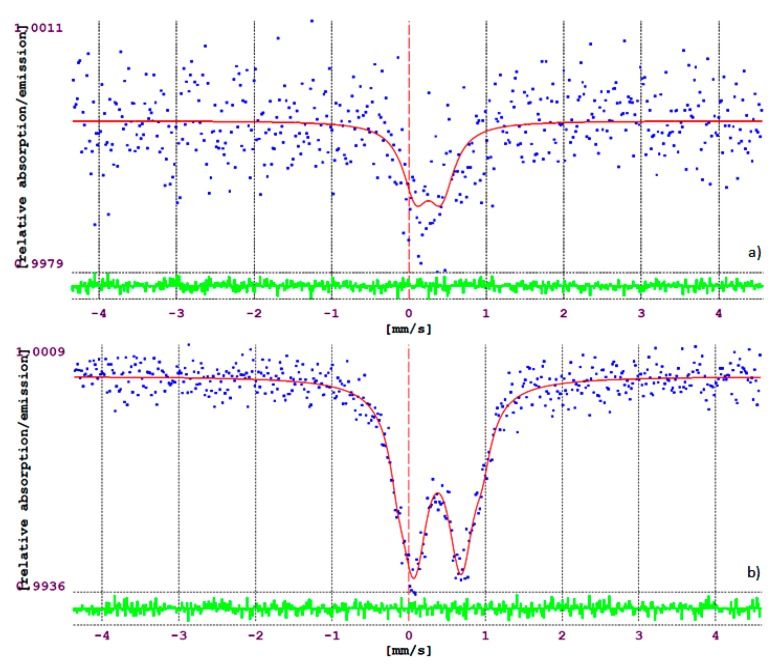
Mössbauer spectra: (**a**) dedoped hybrid system Na-ZSM-5-PT and (**b**) re-doped Na-ZSM-5-PT with FeCl_3_ enriched with radioisotope ^57^Fe.

The potential presence of residual Fe^3+^ and/or Fe^2+^ in a prepared catalyst was verified by X-ray microanalysis and Mössbauer spectroscopy. The Mössbauer spectrum of Fe in the sample of photo-catalyst obtained after thorough dedoping (insulating) by ammonium hydroxide and washing with deionised water, is shown in Figure 4a. Due to the wide dispersion of points, the sample of hybrid photo-catalyst contains only negligible amounts of Fe after dedoping. According to the X-ray microanalysis the sample contained 0.08 wt% Fe and 3.1 wt% S (not shown). Figure 4b shows the Mössbauer spectrum of the hybrid catalyst sample obtained after re-doping with FeCl_3_ enriched with radioisotope ^57^Fe. From the quadruple coupling constant (Δ = 0.61 ± 0.02 mm s^−1^) and from the values of isomer shift (δ = 00:17 ± 0.01 mm s^−1^) it can be stated that the Fe in both samples is mostly present in the oxidation state Fe^3+^. From the fact that the sample after dedoping contains only a negligible amount of Fe and it was not possible to identify the presence of Fe^2+^, it can be concluded that the almost immeasurable amount of Fe cannot be crucially involved in the photoionization of the photochemical reactions that ultimately lead to a reduction of CO_2_.

### 2.2. Detection of Radical Intermediates and Products of CO_2_ Photo-Reduction in the Aquatic Environment; Photo-Reduction Mechanism

In our previous work [19] we studied the formation mechanism of the gaseous products of the CO_2_ reduction in the system of ZSM-5, in which channels oligomers of thiophene were incorporated. The aqueous suspension was saturated with CO_2_ before irradiation; the produced gas phase was continuously collected during the radiation. The presence of gaseous compounds was analyzed by gas chromatography. In the gas mixture only the presence of H_2_ was confirmed. Any organic substance (e.g., methane), that were expected, were not present. We also found that the intensity of the EPR signal (cation radical, polaron) was increased with the irradiation time, which can be attributed to the fact that the excitation stimulated the release of electrons which subsequently let to growth of the PT polarons concentration. The released electron then took part in reactions resulting in the formation of H_2_. The presence of other gaseous products of the CO_2_ photo-reduction was not confirmed.

In our experiments, blank reactions were performed to ensure no hydrocarbon production due to the photo-reduction of CO_2_ and to eliminate the surrounding interference. The first blank experiment was illuminated without the catalyst, the second one was in the dark with the catalyst and CO_2_ under the same experimental conditions, and the third one was illuminated photo-catalyst in the absence of CO_2_. No hydrocarbons were detected in the blank tests.

**Figure 5:**
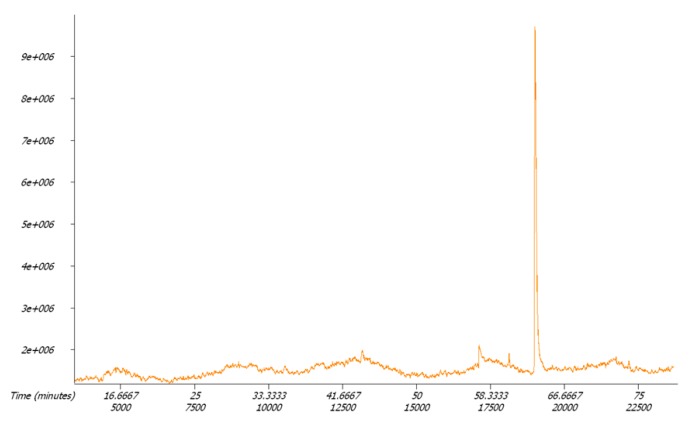
GC chromatogram of CO_2_ reduction product.

The GC chromatogram of CO_2_ reduction products in water obtained after 7.5 h light exposure is shown in Figure 5. The aqueous suspension of the photo-catalyst in this case was bubbled through with CO_2_ during the whole period of irradiation (2 mL min^−1^). The chromatogram shows the CO_2_ photo-reduction product identified by this technique as only one main product—acetic acid (retention time 63.354 min). The presence of this substance was demonstrated using a standard and by measuring the mass spectrum. Based on the knowledge about CO_2_ photo-reduction on TiO_2_ [20], we expected the presence of methanol and therefore we tried to identify methanol by the internal standard method. The methanol gave a retention time of 7.449 min., which was not identified in the sample after 7.5 h exposure. This observation can be explained by the fact that methane likely immediately enters fast, subsequent radical reactions.

**Figure 6:**
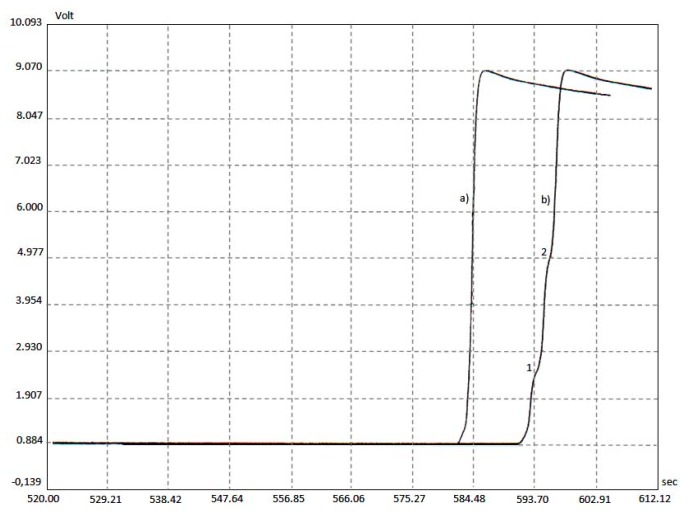
Analysis of CO_2_ photo-reduction products in water after 240 min by isotachophoresis: (**a**) without irradiation; (**b**) 150 W (~60 W m^−2^) metal halogen lamp irradiation with visible light irradiation (240 min). (1—formic acid, 2—acetic acid).

To identify other possible products, the isotachophoresis method was therefore used. Figure 6 shows the isotachophoresis analysis record of the catalyst Na-ZSM-5-PT aquatic suspension bubbled with CO_2_ without and with visible light irradiation (240 min). The analysis of the sample shows, that in the aqueous layer two substances can be identified. One was identified as formic acid with RSH = 0.195 (relative step height) and the other as acetic acid with RSH = 0.492. After 240 min of exposure, in the reaction mixture there was to be found 0.038 µmol L^−1^ formic acid (12.6 µmol g^−1^-cat) and 0.5 µmol L^−1^ acetic acid (166 μmol g^−1^-cat).

**Figure 7:**
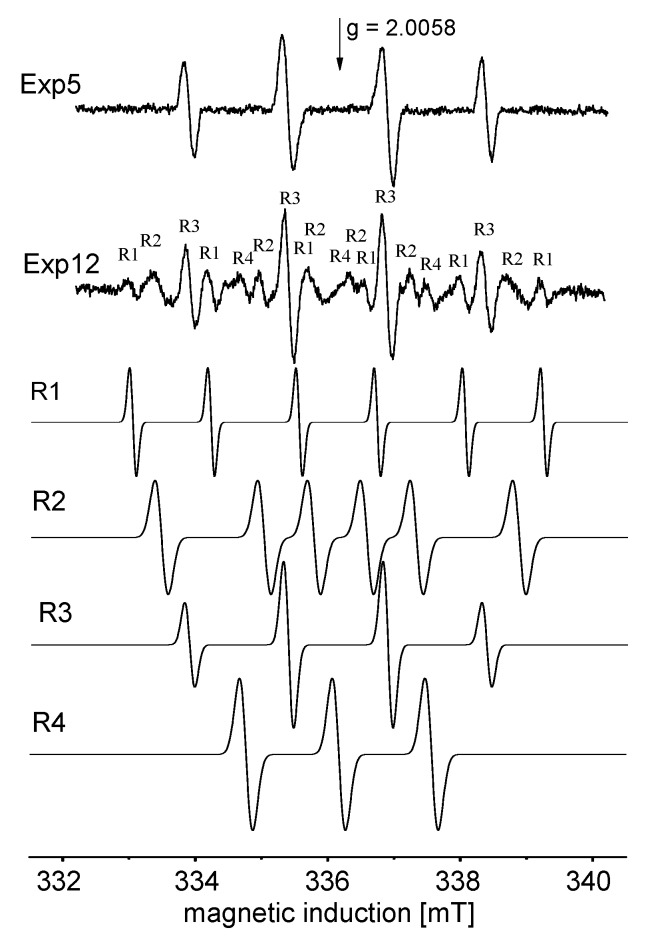
EPR spectra of Na-ZSM5-PT aqueous suspension (bubbled 30 min with CO_2_ before irradiation) after 5 min (Exp5) and 12 min (Exp12) irradiation with a halogen lamp and bubbling with CO_2_ outside of the resonator cavity together with the fitted spectra of spin adducts DMPO-R1, DMPO-R2, DMPO-R3, and DMPO-R4.

EPR spin trapping technique was applied for detection of paramagnetic intermediates. The spin trapping agent DMPO was chosen to follow the formation of paramagnetic species generated upon light exposure. Figure 7 shows the EPR spectra of an aqueous suspension of Na-ZSM-5-PT (bubbled for 30 min with CO_2_ before irradiation) after 5 and 12 min irradiation by the halogen lamp. In the first phase of irradiation only one EPR signal, characterized with spin Hamiltonian parameters A_N_ = 1.501 mT, A_H_ = 1.483 mT, g = 2.0058 was registered (Figure 7, Exp5). This signal belongs to ^•^DMPO–OH spin adduct [21]. During prolongation of time irradiation one can observe production of further radical adducts (Figure 7, Exp.12). The EPR signal after 12 min irradiation consists of four radicals (R1 to R4). By simulation of this spectrum using the EPR-WINSIM 2002, we identified the radical R1 with g = 2.0058 and the hyperfine splitting constants A_N_ = 2.51 mT and A_H_ = 1.18 mT. On the basis of these values, we cannot exactly determine the nature of this radical. We assume that it could be the radical of ^•^CR type. Another radical adduct ^•^DMPO-R2 was identified with g = 2.0058, A_N_ = 1.55 mT, and A_H_ = 2.3 mT. According to the Spin Trap Database [22] the R2 radical could be ^•^DMPO–CH_3_, whose hyperfine splitting constants are in the range of A_N_ = 1.53–1.656 mT and A_H_ = 2.2–2.373 mT. The R2 radical could also be a DMPO adduct with radical ^•^CH_2_OH, for which the hyperfine spitting constants are in the range of A_N_ = 1.545 – 1.62 mT and A_H_ = 2.25–2.3 mT [22]. Or R2 can be a DMPO adduct with radical CH_3_^•^C(H)OH (A_N_ = 1.58–1.588 mT and A_H_ = 2.27–2.28 mT [22,23,24,25]. From the comparison of the ^•^DMPO–R2 hyperfine splitting constants with the above-mentioned literature data, we can assume that this radical is either an adduct DMPO with ^•^CH_3_, ^•^CH_2_OH or CH_3_^•^C(H)OH radical. Consequently, we consider that radical R2 can be a mixture of these three radicals. Radical R3 can be unequivocally assigned to adduct DMPO with ^•^OH radical. The values of g = 2.0058 and coupling constants A_N_ = 1.501 mT and A_H_ = 1.483 mT are very close to the values reported in the literature for such spin adducts [21] with values A_N_ = 1.497 mT and A_H_ = 1.477 mT. Radical R4 with A_N_ = 1.4 mT is probably a product of DMPO decomposition.

Based on currently known facts about the mechanism of CO_2_ photo-reduction on TiO_2_ photo-catalyst [26,27,28,29,30,31,32] and our findings, it is possible, with respect to the registered transitional organic radicals and identified end products, to formulate a photo-reduction mechanism for the hybrid photo-catalysts Na-ZSM-5-PT followed by the partial steps.

**Scheme 1:**
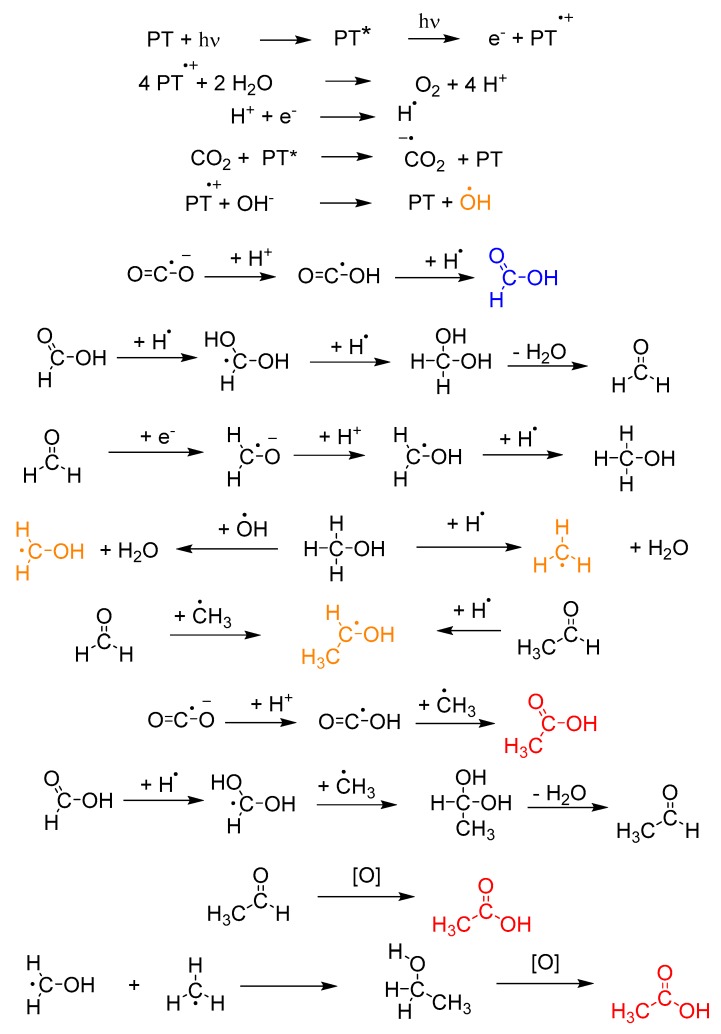
Proposed mechanism of formic acid (blue) and acetic acid formation (red) together with detected radicals (orange).

Our proposed mechanism (Scheme 1) is supported by several other authors [13,14,19,27,28] who discovered the formation of radical intermediates in an aqueous medium in the presence of CO_2_. Based on newly gained knowledge it can be stated that photochemical reduction of CO_2_ is initiated by the release of an electron from the excited state of polythiophene.

The result of this step is the production of a hydrogen radical (H^•^) and an anion-radical of carbon dioxide (CO_2_^•−^). This anion-radical undergoes cascade reactions proposed in Scheme 1 to form the end products (formic acid, acetic acid, etc.). Several radicals (^•^OH, ^•^CH_3_, ^•^CH_2_OH, CH_3_^•^C(H)OH, C^•^, and currently unidentified radical ^•^R1) occur on the surface of the photo-catalyst in the subsequent thermal reactions. The mechanism of radical production in relation to the end product production based on measured EPR spectra can be also explained on the basis of the alternative mechanism discussed in previous works [27,28], proposed for the CO_2_ photo-reduction in an aqueous suspension of TiO_2_. According to the conclusions of these works, carbon radicals (C^•^, measured at low temperature) and atomic oxygen [O] are formed on the surface of the photo-catalyst by photo-reduction of CO_2_, in accordance with the scheme: CO_2_ → CO + O and CO → C^•^ + O. A methyl radical (^•^CH_3_) can be formed also by reaction of C^•^ with a hydrogen radical (H^•^).

The probability, that the chemistry of formation of acetic acid and formic acid takes place according to the proposed mechanism, is confirmed by the fact that, the temperature during the irradiation increased from 22 °C and stabilized at 34 ± 1 °C. This indicates that the subsequent thermal reactions may be influenced by other factors: the catalyst/water ratio, CO_2_/H_2_O ratio, temperature, radiation intensity, and others. Work in this direction is the subject of further studies.

## 3. Experimental

### 3.1. Preparation of Photo-Catalyst

Chemicals used for the photo-catalyst preparation, i.e., anhydrous iron (III) chloride (Fluka), thiophene (Sigma-Aldrich), zeolite Na-ZSM-5 (VÚRUP co., Slovak Republic) and trichloromethane (Merck) were of reagent grade and used without supplementary purification. CO_2_ (Messer Austria GmBH, contains: CO_2_ 99.995%, O_2_ < 10 vpm, N_2_ < 25 vpm, water vapor < 5 vpm, carbohydrates < 1 vpm and CO < 1 vpm) was employed in photo-catalytical reduction tests. 5,5-Dimethyl-1-pyrroline-N-oxide (DMPO, Sigma-Aldrich) was distilled prior to application.

Catalyst Na-ZSM-5-PT was prepared by chemical oxidative polymerization of thiophene with anhydrous FeCl_3_ in the presence of Na-ZSM-5. The used zeolite was of the following composition (molar ratio): Na_2_O:CaO:Al_2_O_3_:SiO_2_ = 0.61:0.34:1.0:43.6. Na-ZSM-5 was treated in a trichloromethane solution of FeCl_3_ (0.25 mol L^−1^) with ultrasonic stirring. After 1 hour, thiophene was added. After 4 h, the product was filtered off, de-doped with NH_4_OH and washed with a sufficient amount of distilled water, dried in air at 20 °C. Prior to removal of reactant residuals, the catalyst was dried in an oven at 105 °C for 2 h.

### 3.2. Characterisation of Photo-Catalyst

X-ray microanalysis and the morphology of photo-catalyst Na-ZSM-5-PT were conducted by scanning electron microscopy with an JXA 840A instrument (JEOL) with an accelerating voltage of 15 kV.

For the study of the overall thermal behavior of photo-catalyst Na-ZSM-5-PT, a thermogravimetric differential thermal analysis (TG-DTA) system (Derivatograph Q 1500D) was used. Sample (100 ± 4 mg) was heated from 30 °C up to 500 °C at a rate of 5 °C min^−1^.

The diffuse reflectance spectra in the UV–VIS region were measured with the fiber optics spectrophotometer system Ocean Optics consisting of an Hi-Res spectrometer HR 4000CG-UV-NIR, UV–VIS light source DH-2000-BAL, and standard reflectance accessory with 45°/45° geometry. Spectra were measured in the region 230–900 nm.

The diffuse reflectance spectra in the range 900–2600 nm were measured with the fiber optics spectrophotometer system Ocean Optics consisting of spectrometer NIR256-2.5, VIS–NIR light source HL-2000-FHSA and standard reflectance accessory with 45°/45° geometry. For each measurement, the detector was calibrated to the blank polymer. In this way, changes in spectral reflectance due to ageing of the substrate were largely excluded.

FTIR spectra were obtained using a Nicolet 6700 FT-IR (Thermo Scientific, USA) FTIR spectrophotometer. The spectra were measured in the range of 600–4000 cm^−1^, sensitivity 4, 40 scans repeated for each analysis. An ATR adapter with a diamond window was used for spectral measurements using the principle of total reflectivity

The Mössbauer spectra were obtained with an Elron-Intertechnique SA-41 cryomagnetic Mössbauer spectrometer. ^57^Co with an activity of about 20 mCi (1 GBq) and a Cr matrix was used as a source. The range of the maximum rate was 10 mm s^−1^. The measured spectra were evaluated according to the NORMOS program.

### 3.3. Photocatalytical Experiment

The photo-catalytical reduction of CO_2_ was carried out in a glass reactor (150 mL) which contained 150 mg modified zeolite and 100 mL of purified water (pH = 5.6). The reactor was bubbled with CO_2_ (2 mL min^−1^) throughout the reaction. The suspension was magnetically stirred during the irradiation by a 150 W metal halogen lamp (Tesla; ≈ 60 W m^−^^2^) passed through a 7 cm water filter for 8 h. After the reaction was finished, the solution was filtered and the filtrate was analyzed by gas chromatography (dynamic headspace GC-TOFMS, sorbent Tenax TA, column DB-FFAP, GC temperature program: 30 °C (20 min)—2 °C min^−^^1^—130 °C—10 °C min^−^^1^—220 °C) equipped with a mass detector. The presence of acids in the sample was evaluated by isotachophoretic analysis (ITP) on Analyser ZKI–02 (Villa Labeco Co., Spišská Nová Ves, Slovakia) using a column-coupling technique. The diameter of the pre-separation column was 0.8 and 0.3 mm for the analytical column. Both columns were equipped with a conductivity detector. Determination of ions was accomplished by the following electrolyte system—solvent: leading A1: 10 mM histidine + HISCL, pH = 6.1, leading A2: 5 mM capric acid + 5 mM histidine.

### 3.4. EPR Spin Trapping Experiment

The EPR spectra were measured by means of EMX Plus X-band EPR spectrometer with a High Sensitivity Probe-head (Bruker). An aqueous suspension of Na-ZSM-5-PT (1.5 mg mL^−1^, 400 μL) mixed with 300 μL of DMPO solution (0.212 M) was saturated by CO_2_ using a mild gas stream. The prepared system was photo-excited by the radiation of a 150 W metal halogen lamp through a 7 cm water filter outside the EPR resonator. After 5 respective 12-minute exposure the suspension was immediately transferred into the small quartz flat cell (WG 808-Q, Wilmad-LabGlass, optical cell length 0.045 cm) and EPR spectra were recorded.

## 4. Conclusions

A new hybrid photo-catalyst on the basis of a zeolite Na-ZSM-5 was synthesized. On the surface of the crystals was an in situ deposited layer of organic polymer semiconductor—polythiophene. Polymerization of thiophene was carried out by oxidative polymerization with FeCl_3_. To ensure the “uniformity” of the crystal surface coverage, polymerization was conducted under ultrasonic stirring participation. The photo-catalyst was applied to the photo-reduction of CO_2_ using VIS light. In this way synthesized hybrid photo-catalyst showed high thermal stability in an oxygen environment. The photo-catalyst is not soluble in any ordinary organic solvent. The advantage of such a photo-catalyst is the fact that the polythiophene strongly absorbs light radiation up to ca 650 nm, with the absorption edge encroaching into NIR, which allows the use of spectral favorable sources for CO_2_ reduction. Analysis of the products resulting from the reduction of CO_2_ showed the presence of two products, acetic acid and lesser concentrations of formic acid. This fact allows the use of the main product—the acetic acid—for the preparation of ammonium acetate, which has widespread use in chemical practice, particularly as a raw material for various syntheses and pH adjustment. In future, it may be possible to use this method (e.g., by introducing nitrogen atom to the molecule through ammonium acetate) for the production of specialty value added chemicals. The mechanism of reaction product formation was suggested.

## Supplementary Materials

The following are available online, Figure S1: Left: Pure Na-ZMS-5 before reaction. Right: Photo-catalyst prepared without participation of ultrasound. Small amount of FeCl_3_ (~ 0.05 mol L^−1^) was present in reaction mixture, Figure S2: Left: Pure Na-ZMS-5 before reaction. Right: Photo-catalyst prepared with participation of ultrasound. FeCl_3_ (0.25 mol L^−1^) was present in reaction mixture, Figure S3: Left: Pure Na-ZMS-5 before reaction. Right: Photo-catalyst prepared with participation of ultrasound doped with FeCl_3_ (0.55 mol L^−1^), Figure S4. FT-IR spectrum of photo-catalyst before reaction, Figure S5. FT-IR spectrum of photo-catalyst begore reaction.
