# Supplementary material for: Photo-Reduction of CO2 by VIS Light on Polythiophene-ZSM-5 Zeolite Hybrid Photo-Catalyst
Source: Molecules. 2019 Mar 12;24(5):992. doi: 10.3390/molecules24050992 (PMC6429064; doi:10.3390/molecules24050992)
Supplement: Supplementary file 1 [file molecules-24-00992-s001.pdf]

molecules

Photo-Reduction of CO_2 by VIS Light on Polythiophene-ZSM-5 Zeolite Hybrid Photo-Catalyst

Jana Kianička [1], Gabriel Čík [1,*], František Šeršeň [2,*], Ivan Špánik [3], Robert Sokolík [2] and Juraj Filo [2,*]

[1] Department of Environmental Engineering, Faculty of Chemical and Food Technology, Slovak University of Technology in Bratislava, Radlinského 9, SK-812 37 Bratislava, Slovakia; jana@kianicka.name
[2] Institute of Chemistry, Faculty of Natural Science, Comenius University in Bratislava, Ilkovičova 6, SK-842 15 Bratislava, Slovakia; robert.sokolik@uniba.sk
[3] Department of Analytical Chemistry, Faculty of Chemical and Food Technology, Slovak University of Technology in Bratislava, Radlinského 9, SK-812 37 Bratislava, Slovakia; ivan.spanik@stuba.sk
* Correspondence: gabriel.cik@stuba.sk (G.Č.); f.sersen@gmail.com (F.Š.); juraj.filo@uniba.sk (J.F.)

Electronic Supplementary information

(ESI)

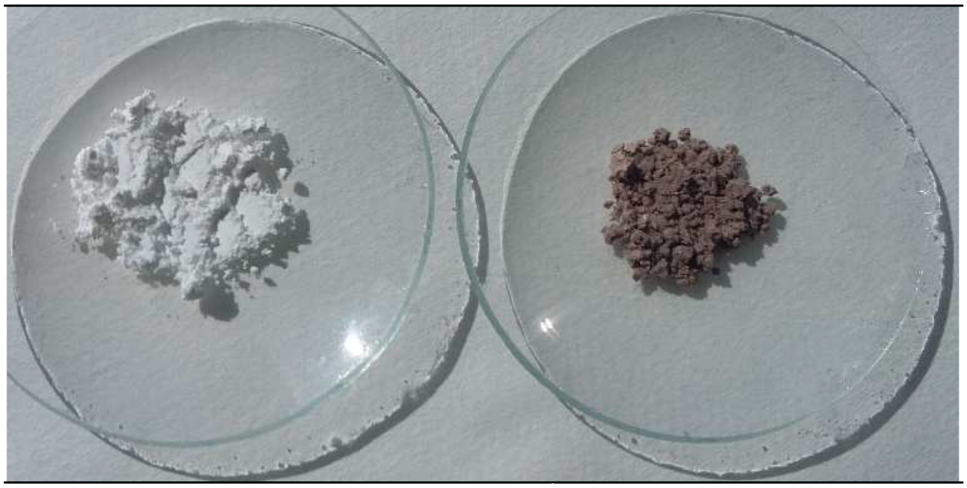

Figure 1. Left: Pure Na-ZMS-5 before reaction. Right: Photo-catalyst prepared without participation of ultrasound. Small amount of FeCl3 (~ 0.05 mol L^{-1}) was present in reaction mixture.

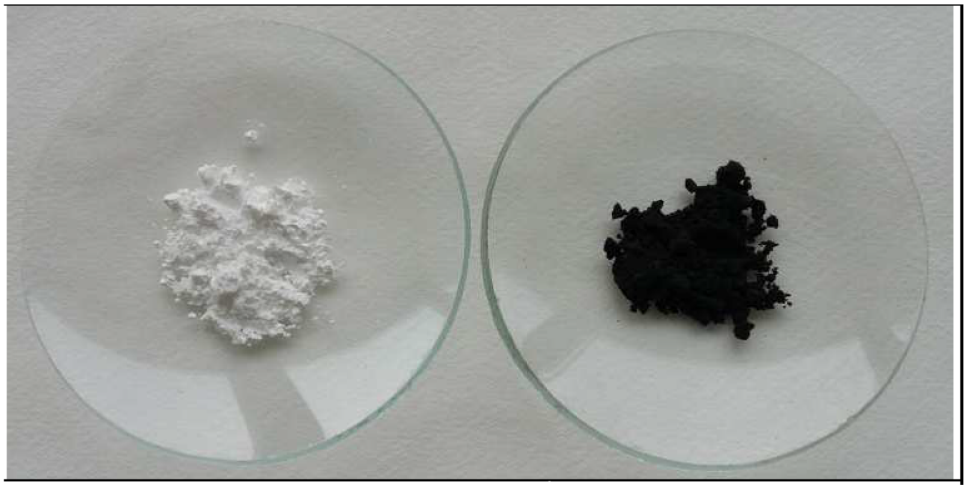

Figure S2. Left: Pure Na-ZMS-5 before reaction. Right: Photo-catalyst prepared with participation of ultrasound. $FeCl_3$ (0.25 mol L^{-1}) was present in reaction mixture.

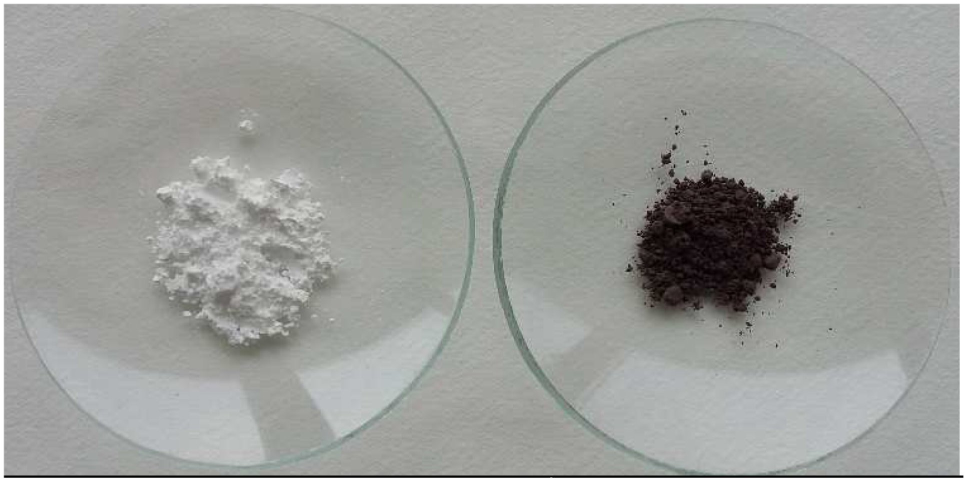

Figure S3. Left: Pure Na-ZMS-5 before reaction. Right: Photo-catalyst prepared with participation of ultrasound doped with FeCl3 (0.55 mol L^{-1}).

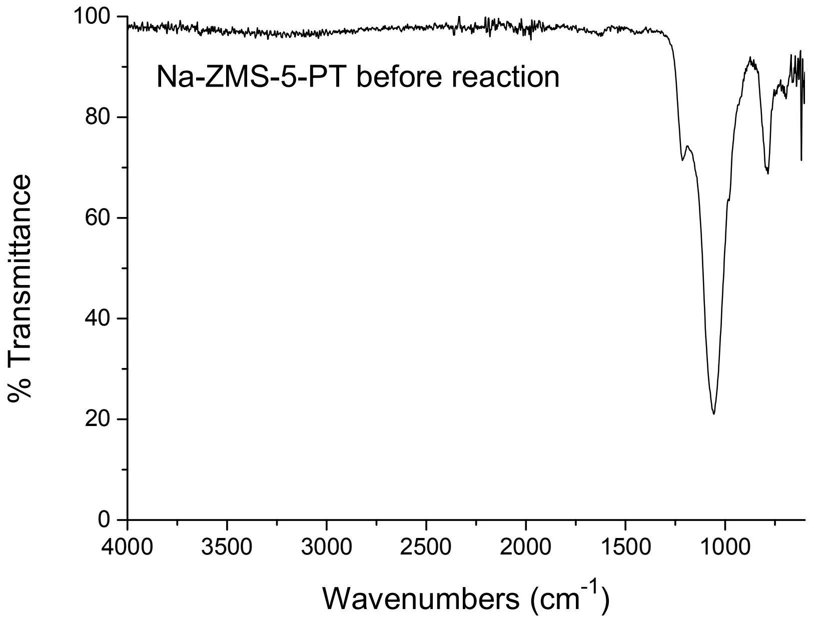

Figure S4. FT-IR spectrum of photo-catalyst before reaction.

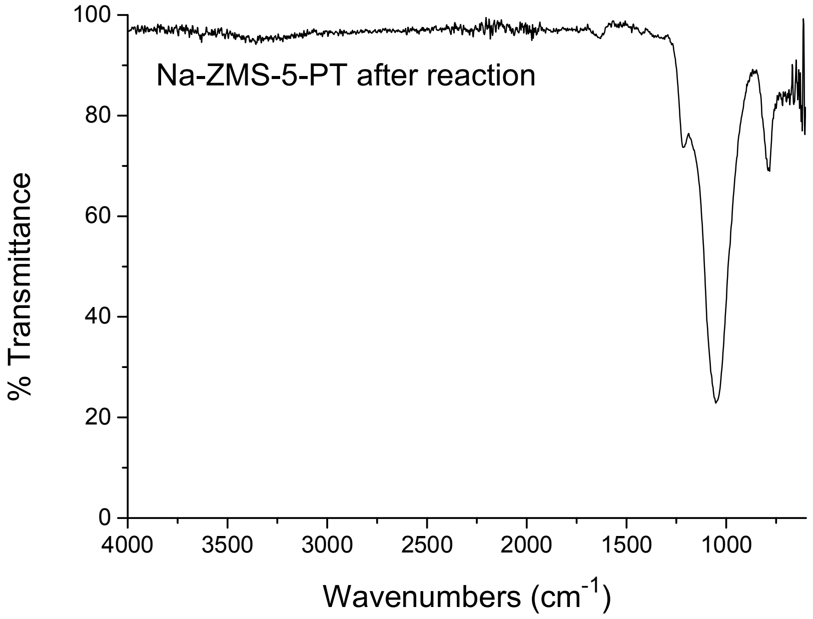

Figure S5. FT-IR spectrum of photo-catalyst after reaction.
